# The C-terminal lectin-like domain modulates the substrate specificity and transglycosylation activity of rice β-galactosidase1 (*Os*BGal1)

**DOI:** 10.7717/peerj.21066

**Published:** 2026-04-09

**Authors:** Sunaree Choknud, Thipwarin Rimlumduan, Surapoj Sanram, Jenis Chiawitthayanan, Wipa Suginta, James R. Ketudat Cairns

**Affiliations:** 1 School of Chemistry, Institute of Science, Suranaree University of Technology, Nakhon Ratchasima, Thailand; 2 Center for Biomolecular Structure, Function and Application, Suranaree University of Technology, Nakhon Ratchasima, Thailand; 3 Department of Applied Biology, Faculty of Science and Liberal Arts, Rajamangala University of Technology Isan, Nakhon Ratchasima, Thailand; 4 School of Biomolecular Science and Engineering, Vidyasirimedhi Institute of Science and Technology, Rayong, Thailand

**Keywords:** Glycoside hydrolase, Accessory domain, Glycosidase, *Pichia pastoris*, Glycosylation

## Abstract

**Background:**

β-galactosidases (EC 3.2.1.23) are glycosidases that release nonreducing terminal β-d-galactosyl residues from saccharides or glycosides. In plants, most characterized β-galactosidases belong to glycoside hydrolase family 35 (GH35) and function in cell wall remodeling. Over half of plant GH35 enzymes contain a C-terminal lectin-like domain absent in GH35 enzymes from other kingdoms, but its function remains unclear. In this work, we investigate the role of the C-terminal lectin-like domain by the effect of its deletion in rice β-galactosidase 1 (*Os*BGal1) on its enzymatic function.

**Methods:**

We expressed *Os*BGal1 and a truncated variant lacking the C-terminal domain (*Os*BGal1ΔCter) in *Pichia pastoris*. Both enzymes were purified to homogeneity, with *Os*BGal1ΔCter exhibiting a higher level of N-glycosylation than the full-length enzyme. Kinetic analysis of *p*NP-glycosides evaluated substrate preferences. Molecular dynamics (MD) simulations of the predicted structures were used to visualize the effect of the C-terminal lectin-like domain.

**Results:**

*Os*BGal1 showed high activity toward *p*NP-β-d-galactopyranoside and negligible activity toward alternative substrates, except for *p*NP-β-d-fucopyranoside. In contrast, *Os*BGal1ΔCter exhibited a 20-fold reduction in catalytic efficiency toward *p*NP-β-d-galactopyranoside but acquired significant activity toward *p*NP-β-d-glucopyranoside and higher activity for *p*NP-β-d-fucopyranoside. Kinetic analysis confirmed this broadened substrate profile, with the truncated enzyme showing higher turnover on glucoside, fucoside, and xyloside substrates. Functional assays demonstrated that *Os*BGal1 catalyzes transglycosylation, whereas *Os*BGal1ΔCter lost this activity. The predicted structures of *Os*BGal1 showed that the C-terminal domain is more than 30 Å from the active site, and MD simulations validated these structures by demonstrating their stability. This study reveals that removal of the C-terminal lectin-like domain of *Os*BGal1 alters substrate specificity and reduces transglycosylation activity, which may provide a basis for engineering GH35 enzymes with customized catalytic properties.

## Introduction

The β-galactosidases (EC 3.2.1.23) that have been classified by the carbohydrate-active enzymes (CAZy) database to date fall into the amino acid-sequence-based glycoside hydrolase (GH) families 1, 2, 35, 42, 50, and 59 (Lombard, Henrissat & Garron, 2024). β-galactosidases cleave terminal β-d-galactosyl residues from oligosaccharides, polysaccharides, and glycoconjugates. They are widely distributed across bacteria, fungi, plants, and animals, reflecting their fundamental role in carbohydrate metabolism (Saburi et al., 2023; Trainotti et al., 2001; Morita et al., 2009).

Characterized plant β-galactosidases belong to GH family 35 (GH35). Comparative studies have revealed that plants harbor multiple GH35 β-galactosidases, each with distinct isoforms expressed during development (Ahn et al., 2007; Tanthanuch et al., 2008). Together, these studies underscore the conserved catalytic core of GH35 enzymes, while highlighting their functional diversification through gene expansion, differential expression, and the acquisition of accessory domains. In apples, strawberries, and tomatoes, specific isoforms correlate with fruit softening and texture (Smith & Gross, 2000; Trainotti et al., 2001; Gwanpua et al., 2014). Although many studies focus on fruit ripening, β -galactosidase expression has also been correlated with asparagus softening at harvest, as well as with flower opening and senescence, consistent with these enzymes acting in cell wall modifications of agricultural importance.

Based on protein sequence comparison of GH35 sequences encoded in the genomes of Arabidopsis, rice (*Oryza sativa*), and the bryophyte moss *Physcomitrella patens*, plant GH35 enzymes have been split into subfamilies a1, a2, a3, a4, a5, b, c1, c2, and d, (Ahn et al., 2007; Tanthanuch et al., 2008). Among these, subfamily d is more closely related to animal β-galactosidase than to the enzymes in the other plant subfamilies. All the Arabidopsis subfamily a1 members (*At*BGAL 1, 2, 3, 4, 5, and 12) have been expressed in *Pichia pastoris* and the proteins characterized and shown to release galactose from galactobiose, galactotriose, and cell wall pectin, but not xyloglucans (Ahn et al., 2007; Gantulga et al., 2008; Gantulga et al., 2009). Similarly, *At*BGAL6 (also called MUM2, a subfamily c2 member) was found to modify pectin galactosyl sidechains, and its absence resulted in less seed mucilage extrusion during seed germination (Dean et al., 2007; Macquet et al., 2007). In contrast, *At*BGAL10, a subfamily a5 member, was found to remove galactosyl units from xyloglucan sidechains, and its absence resulted in abnormal xyloglucan repeated units and shorter siliques and sepals (Sampedro et al., 2012). Most of these *Arabidopsis* GH35 enzymes have been localized to the cell wall, supporting their role in cell wall development (Chandrasekar & Ra, 2016).

In rice, fifteen GH35 genes have been identified (Tanthanuch et al., 2008), although grasses like rice have type II cell walls with less pectin and xyloglucan. *Os*BGal1 and *Os*BGal2 were both found to hydrolyze and transglycosylate β-galactosides (Chantarangsee et al., 2007). *Os*BGal1 also had activity on β-galactobioses, with the highest activity on β-1,3-, followed by β-1,4- and β-1,6- galactobiose, and also released galactose from larch wood arabinogalactan. Given the high expression of *Os*BGal in rapidly expanding tissues, including 3-day-old seedling shoot, stem apical meristem, initiating panicle and flower (Chantarangsee et al., 2007; Tanthanuch et al., 2008), this suggests a role for *Os*BGal1 in remodeling the cell wall in rapidly growing tissues *via* its action on arabinogalactans. Another rice isoenzyme, *Os*BGal13, was also shown to hydrolyze β-1,3-, β-1,6-, and β-1,4-galactobiose disaccharides (in order of activity) and to exhibit strong transglycosylation of β-1,4-galactobiose (Tanthanuch et al., 2008). It hydrolyzed *p*-nitrophenyl-α-l-arabinoside with 30% of its activity on *p*-nitrophenyl-β-d-galactoside, suggesting it might also act on both types of monosaccharide residues in arabinogalactan, but no release of arabinose or galactose from larch wood arabinogalactan was detected. More recently, the gene encoding *Os*BGal9, an isoenzyme more closely related to animal β-galactosidases than other rice isoenzymes, was identified as being regulated by the stress-induced transcription factor SPOTTED-LEAF7, and its expression correlated with resistance to abiotic and biotic stress (Hoang et al., 2023). *Os*BGal9 was active as a β-galactosidase, but no differences in galactose levels were seen in knockdown and overexpression rice lines. Nonetheless, staining of arabinogalactan in arabinogalactan proteins increased in knockout plants and decreased in overexpression plants, suggesting a role in arabinogalactan remodeling involved in stress resistance.

**Figure 1:**
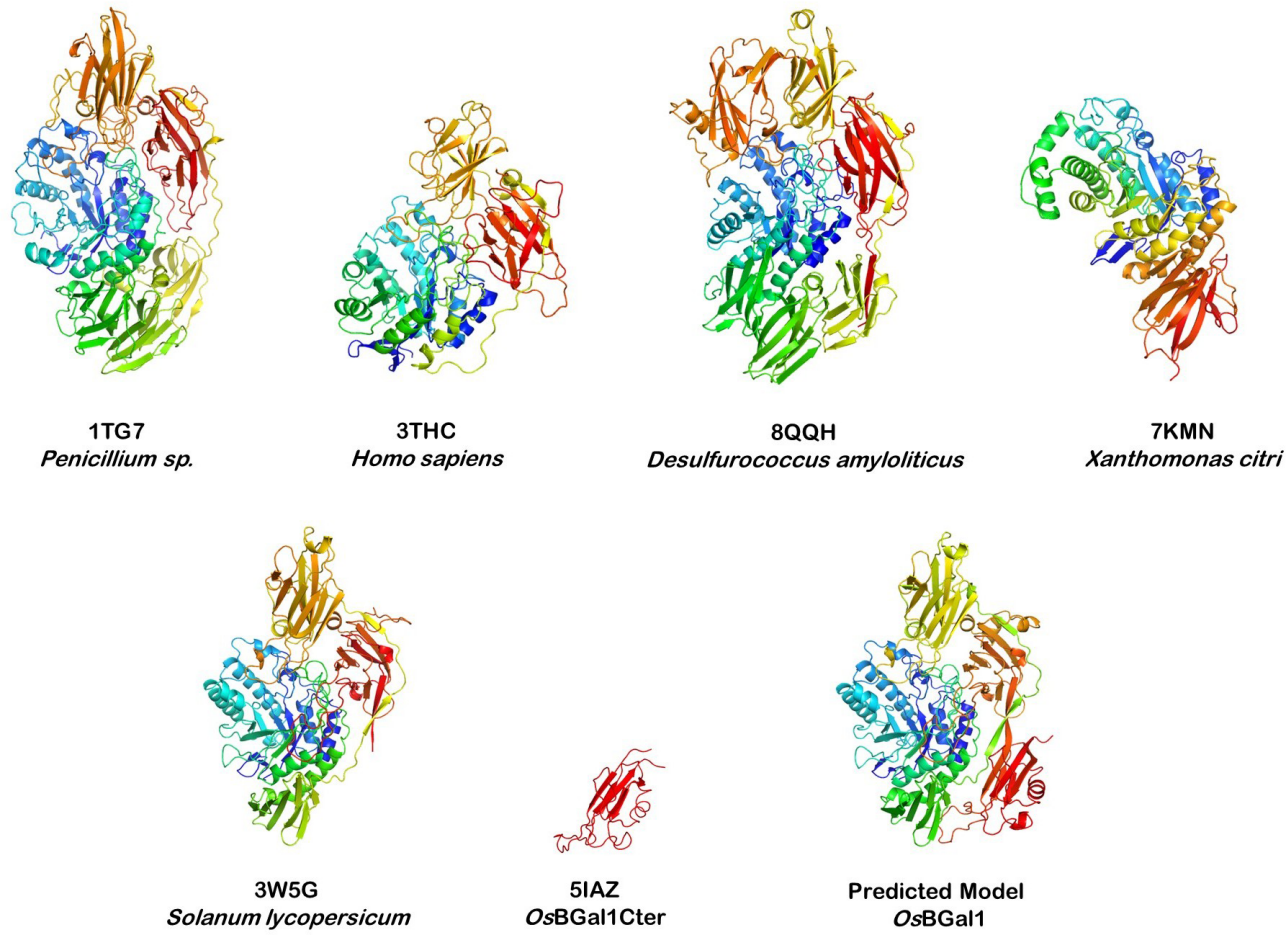
Structural comparison of GH35 β-galactosidases from different kingdoms. Top: Selected GH35 structures from fungi, human, archaea, and bacteria, including *Penicillium* sp. (PDB: 1TG7), *Homo sapiens* (PDB: 3THC), *Desulfurococcus amylolyticus* (PDB: 8QQH), and *Xanthomonas citri* (PDB: 7KMN). Bottom: the crystal structure of tomato β-galactosidase TBGal4 (PDB: 3W5G) (left), NMR structure of the isolated C-terminal lectin-like domain of *Os*BGal1 (PDB: 5IAZ) (center), and predicted full-length model of rice *Os*BGal1 (right). All the full-length protein structures share a conserved N-terminal (β/α)_8_ TIM-barrel catalytic domain (blue/green) but differ in the number and arrangement of β-sandwich domains (orange/red). Of note, none of the experimental full-length GH35 structures contain a C-terminal lectin-like extension, a plant-specific feature highlighted in red in the *Os*BGal1 model and 5IAZ domain structure.

GH35 enzymes share a conserved (β/α)_8_ TIM-barrel catalytic core (Henrissat & Davies, 1997), which houses two critical acidic residues that act as the nucleophile and proton donor in the double-displacement mechanism typical of retaining glycosidases (Sinnott, 1990), as well as accessory β-sandwich domains, as shown in Fig. 1. For example, fungal enzymes such as *Penicillium* sp. β-galactosidase contain six domains, while human and bacterial homologs typically include two or three (Rojas et al., 2004; Ohto et al., 2012). The first structure of a plant GH35 enzyme, tomato β-galactosidase 4 (TBGal4), was solved and found to possess the conserved catalytic barrel and three β-sandwich domains (PDB: 3W5G) (Eda et al., 2016). Despite their conserved catalytic machinery, GH35 enzymes exhibit functional diversity and are adapted to distinct biological contexts.

Many plant β-galactosidases carry a unique C-terminal lectin-like domain not found in any of these structures (Trainotti et al., 2001; Ahn et al., 2007; Tanthanuch et al., 2008). The lack of a complete structure, including the plant-specific C-terminal extension, leaves a gap in our understanding of the structural and functional significance of this unique feature. The C-terminal lectin-like domain structure of rice *Os*BGal1 has been determined by NMR (Rimlumduan et al., 2016); however, the full-length structure remains unverified. Moreover, although this domain was initially speculated to play a role in carbohydrate binding (Trainotti et al., 2001), no carbohydrate binding activity was detected in the isolated domain (Rimlumduan et al., 2016). *Os*BGal1, which contains this domain, and *Os*BGal2, which lacks this domain, were both found to hydrolyze β-galactosides specifically but not β-glucosides (Chantarangsee et al., 2007). Similarly, *Arabidopsis* GH35 subfamily 1a members *At*BGAL1 and *At*BGAL3 contain this C-terminal domain, while *At*BGAL2, *At*BGAL4, *At*BGAL5, and *At*BGAL12 do not, but they all were found to have similar activity (Gantulga et al., 2009). However, *At*BGAL1 and 3 had weak activity on alkali-solubilized cell walls, which was not detected in the others. No lectin-like activity could be detected for either *At*BGAL1 or *At*BGAL3. *At*BGAL6 and *At*BGAL10 also lack the C-terminal domain, so nearly all *Arabidopsis* GH35 enzymes without the C-terminal domain have been characterized and are found with divergent phylogeny and functions, while only two of the ten enzymes with the domain have been characterized (Ahn et al., 2007). As such, no consistent functional correlation has yet emerged for the C-terminal domain.

The functional contribution of the plant β-galactosidase C-terminal lectin-like domain to enzyme function remains poorly understood. Accordingly, in this study, we examine the functional influence of the C-terminal lectin-like domain in *Os*BGal1. We expressed and purified both full-length *Os*BGal1 and a variant without the C-terminal domain (*Os*BGal1ΔCter) in *Pichia pastoris* (*P. pastoris*) and used substrate specificity and transglycosylation assays to show how that domain shapes galactoside selectivity and catalytic versatility in GH35 β-galactosidases.

## Materials & Methods

### Chemicals

The substrates *p*-nitrophenyl (*p*NP)-β-d-galactopyranoside (*p*NPGal), *p*NP-β-d-glucopyranoside (*p*NPGlc), *p*NP-α-L-arabinopyranoside (*p*NPAra), *p*NP-β-d-fucopyranoside (*p*NPFuc), *p*NP-β-d-xylopyranoside (*p*NPXyl) were acquired from Sigma-Aldrich (St. Louis, MO, USA). Methanol, ethanol, propanol, isopropanol, butanol, glycerol, and ethylene glycol were purchased from CARLO ERBA Reagents (Barcelona, Spain).

### Cloning of *Os*BGal1 and *Os*BGal1ΔCter

A gene encoding *OsBGal1* (GenBank: AAM34271.1, gene locus *Os03g0165400*) optimized for expression in *P. pastoris* (DDBJ: LC898194) was produced by GenScript Corporation (Piscataway, NJ, USA) and provided in the pUC57 vector (Supplementary Information). The optimized *Os*BGal1 cDNA was inserted into the *Pst*I and *Xba*I sites in the pPICZαBNH8 plasmid (Toonkool et al., 2006). The cDNA fragment encoding the *Os*BGal1ΔCter was generated by PCR using pPICZαBNH8/*OsBGal1* as a template with the *OsBGal1*Δ*Cter* Forward primer (5′-CCGCTGCAGTTACTTACGATAAGAAAGC-3′) and Reverse primer (5′GGTCTAGATTATTTAGCCAAAGCAATCTTAG-3′). Amplification was performed by incubating at 95 °C for 5 min, followed by 30 cycles of 95 °C for 30 s, 60 °C for 30 s, and 72 °C for 4 min, then a final extension at 72 °C for 10 min using *Pfu* polymerase. The PCR product was ligated into the *Pst*I and *Xba*I sites in the pPICZαBNH8 plasmid then transformed into competent DH5α cells. The C-terminal domain removed in *Os*BGal1ΔCter corresponds to amino acid residues 734–851, comprising 118 amino acids. A schematic representation of the expression plasmids is provided in Fig. S1. The transformants were selected on agar plates containing 25 µg/ml zeocin. The products were confirmed by DNA sequencing (Macrogen, Seoul, Korea). The pPICZαBNH8/*OsBGal1* and pPICZαBNH8/*OsBGal1*Δ*Cter* plasmids were then digested with *Sac*I and transformed into *P. pastoris* strain SMD1168H by electroporation. Clones were selected on agar plates containing 250 µg/ml zeocin as described in the *Pichia* Manual (Invitrogen, Carlsbad, Calif., USA) and screened for protein expression by a β-galactosidase activity assay, with 20 µL of culture medium, 2 mM *p*NP-glycosides in 50 mM sodium acetate, pH 5.0, in 140 µL reactions incubated at 30 °C for 20 min. The reactions were stopped by adding 70 µL of 2.0 M Na_2_CO_3_, and the absorbance at 405 nm of the released *p*-nitrophenolate (*p*NP) was measured.

### Expression and purification of *Os*BGal1 and *Os*BGal1ΔCter

The *Os*BGal1 and *Os*BGal1ΔCter expression clones with the highest β-galactosidase activity were cultured in buffered glycerol-complex medium (BMGY) for 24 hr at 28 °C while shaking at 200 rpm. The cells were collected by centrifugation at 3,000 ×*g* for 20 min at 20 °C, then resuspended in buffered methanol-complex medium (BMMY) at 20 °C while the culture was incubated in a shaking incubator at 200 rpm. Protein expression was induced by adding 1% (v/v) methanol to the culture medium daily for 3 days. The cells were removed from the media by centrifugation, then adjusted to 7.5 with 1.0 M Na_2_PO_3_, and 200 mL of the culture medium was loaded onto a 5-mL immobilized metal-ion affinity chromatography (IMAC) column charged with Co^2+^ from cobalt chloride. The column was washed with 3 column volumes (CV) of 20 mM Tris–HCl, pH 8.0, 150 mM NaCl (EQ buffer), and 3 CV of 5 mM imidazole in EQ buffer, then the proteins were eluted with 3 CV of 100 mM imidazole in EQ buffer, collecting 5-ml fractions. The fractions were assayed for β-galactosidase activity and analyzed by SDS-PAGE. Apparently pure fractions containing β-galactosidase activity were pooled and concentrated by centrifugal filtration using a 30 kDa molecular weight cutoff membrane. The proteins were purified by size-exclusion chromatography (SEC) on a Superdex S200 Increase 10/300 GL column in EQ buffer at a flow rate of 0.5 mL/min. The fractions containing β-galactosidase activity were analyzed on SDS-PAGE, and the apparently pure fractions were pooled and concentrated by centrifugal filtration, then kept at −80 °C.

### Substrate specificity of *Os*BGal1and *Os*BGal1ΔCter

The substrate specificities were assayed with 5 ng of the enzyme and 1.0 mM *p*NP-glycosides in 50 mM sodium acetate, pH 5.0, at 30 °C for 20 min. The 140 µL reactions were stopped by adding 70 µL of 2.0 M Na_2_CO_3_, and the absorbance of the released pNP at 405 nm was measured. The concentration was then calculated based on a *p*NP standard curve.

### Determination of the pH and temperature optimum and stability of *Os*BGal1 and *Os*BGal1ΔCter

The pH-activity profiles of *Os*BGal1 and *Os*BGal1ΔCter were determined in 140 µL reactions using *p*NPGal for *Os*BGal1 and *p*NPGlc for *Os*BGal1ΔCter at a substrate concentration of 2.0 mM in 50 mM McIlvaine citrate phosphate buffers (McIlvaine, 1921) with pH values between 2.5 and 8.0 at 0.5-pH-unit increments. The reactions were incubated at 30 °C for 20 min and then stopped with 70 µL of 2 M Na_2_CO_3_. The *p*NP product was measured by its absorbance at 405 nm. Temperature dependence was assessed at 10−70 °C in reactions conducted in 50 mM sodium acetate, pH 5.0, for 20 min. Then, the reactions were stopped with 70 µL of 2 M Na_2_CO_3_, and the absorbance at 405 nm was measured. Enzyme stability over time was assessed by incubating samples at selected temperatures (10–60 °C) in EQ buffer for varying periods (0.5, 1, 3, 6, 12, and 24 hr). The reactions were then incubated at 30 °C for 20 min in 50 mM sodium acetate, pH 5.0. Then, the reactions were stopped by adding 70 µL of 2 M Na_2_CO_3_, and the absorbance at 405 nm was measured to quantify the *p*NP released.

### Determination of *Os*BGal1 and *Os*BGal1ΔCter enzyme kinetic parameters

The kinetic parameters of *Os*BGal1 and *Os*BGal1ΔCter were evaluated from triplicate reactions of hydrolysis of *p*NPGal, *p*NPFuc, and *p*NPGlc. The reactions were carried out in a 140 µL volume with 7.5 ng of the enzyme in 50 mM sodium acetate, pH 5.0, at 30 °C for 20 min. Then, the reactions were stopped by adding 70 µL of 2 M Na_2_CO_3_, and the absorbance at 405 nm was measured to quantify the *p*NP released. The substrate concentrations used ranged from one-third to three times the apparent *K*_m_, or beyond, except for *p*NPFuc with *Os*BGal1, for which the *K*_m_ was too high to reach the upper range. The *K*_m_ and *V*_max_ values were determined by non-linear regression analysis of Michaelis–Menten plots with Grafit 5.0 (Erithacus Software, Horley, Surrey, U.K.) and compared to parameters from linear plots of (initial velocity)^−1^ *versus* [substrate]^−1^ (Lineweaver-Burk plots) and [substrate] (initial velocity)^−1^ *versus* [substrate] (Hanes-Woolf plots) to verify similar values. Apparent catalytic rate constant (*k*_cat_) values are calculated by dividing the limiting velocity (*V*_max_) by the amount of enzyme protein in the reaction.

### Transglycosylation activity of *Os*BGal1 and *Os*BGal1ΔCter

The transglycosylation activities were tested using *p*NPGal, *p*NPFuc, and *p*NPGlc as glycosyl donors, and methanol, ethanol, *n*-propanol, isopropanol, butanol, glycerol, and ethylene glycol as acceptors. The transglycosylation reactions contained 5.0 mM glycosyl donors in a 100 µL volume with 1.0 µg of the enzyme in 50 mM sodium acetate, pH 5.0, at 30 °C for 1 and 12 hr. After stopping the reactions by heating at 90 °C for 5 min, as previously described (Choknud et al., 2023), they were spotted onto silica gel 60 F_254_ TLC plates. The TLC plates were developed in a solvent system of butanol:acetic acid:water (2:1:1, v/v/v), then sprayed with 10% sulfuric acid in ethanol and heated at 120 °C until glycoside compounds appeared as dark spots.

### Statistical analysis

We evaluated the significance of differences in activity between *Os*BGal1ΔCter and *Os*BGal1 by the *t*-test implemented in MedCalc Comparison of means (MedCalc Software Ltd. Comparison of means. https://www.medcalc.org/en/calc/comparison_of_means.php (Version 23.4.5; accessed January 3, 2026)). In the cases where activity with *Os*BGal1 was undetectable, the significance of the *Os*BGal1ΔCter was calculated with Test for one mean in the MedCalc Software.

### Molecular dynamics simulation

The initial three-dimensional structure of *Os*BGaI1 was generated with SWISS-MODEL using TBGal4 (3W5G) as a template (Waterhouse et al., 2018; Eda et al., 2016). AlphaFold 3 models were also generated, but some active-site amino acid residues were out of position for catalysis, so the SWISS-MODEL structural model was used for further work. A truncated construct lacking the C-terminus (*Os*BGaI1ΔCter) was prepared by manually deleting the missing residues to investigate the role of the terminal region. Both the full-length and truncated models were submitted to the CHARMM-GUI web server for system construction with the CHARMM36m force field (Huang et al., 2017; Jo et al., 2008). Each protein system was solvated in a TIP3P water box with periodic boundary conditions and neutralized with counter-ions (Jorgensen et al., 1983). Additionally, independent systems were generated by replacing neutralizing ions with 150 mM KCl to assess ion-dependent effects. All simulations were performed using GROMACS 2023 (Abraham et al., 2015). The steepest descent algorithm was applied for energy minimization until a maximum force of less than 1,000 kJ mol^−^^1^ nm^−^^1^ was reached (Press et al., 2007). Positional restraints were applied on heavy atoms with force constants of 400 kJ mol^−^^1^ nm^−^^2^ for the backbone and 40 kJ mol^−^^1^ nm^−^^2^ for side chains (Huang et al., 2017). Subsequent equilibration was carried out in two stages: (i) 125 ps of NVT dynamics with a 1 fs integration step, employing the velocity-rescaling thermostat at 303 K (Bussi, Donadio & Parrinello, 2007); and (ii) 250 ps of NPT equilibration under isotropic pressure coupling with the C-rescale barostat (*τ*p = 5 ps, compressibility = 4.5  × 10^−^^5^ bar^−^^1^) at 1 bar (Parrinello & Rahman, 1981). Production simulations were conducted for 50 ns with a 2 fs timestep, using the Verlet cutoff scheme (Páll & Hess, 2013). Long-range electrostatic interactions were treated using the particle-mesh Ewald (PME) method (Essmann et al., 1995), and van der Waals interactions were smoothly switched off between 1.0 and 1.2 nm (Huang et al., 2017). Bond constraints on hydrogen atoms were applied with the LINCS algorithm (Hess et al., 1997). Coordinates were recorded every 100 ps, and trajectory compression was set to 50 ps intervals.

## Results

### Predicted structure of *Os*BGal1

**Figure 2:**
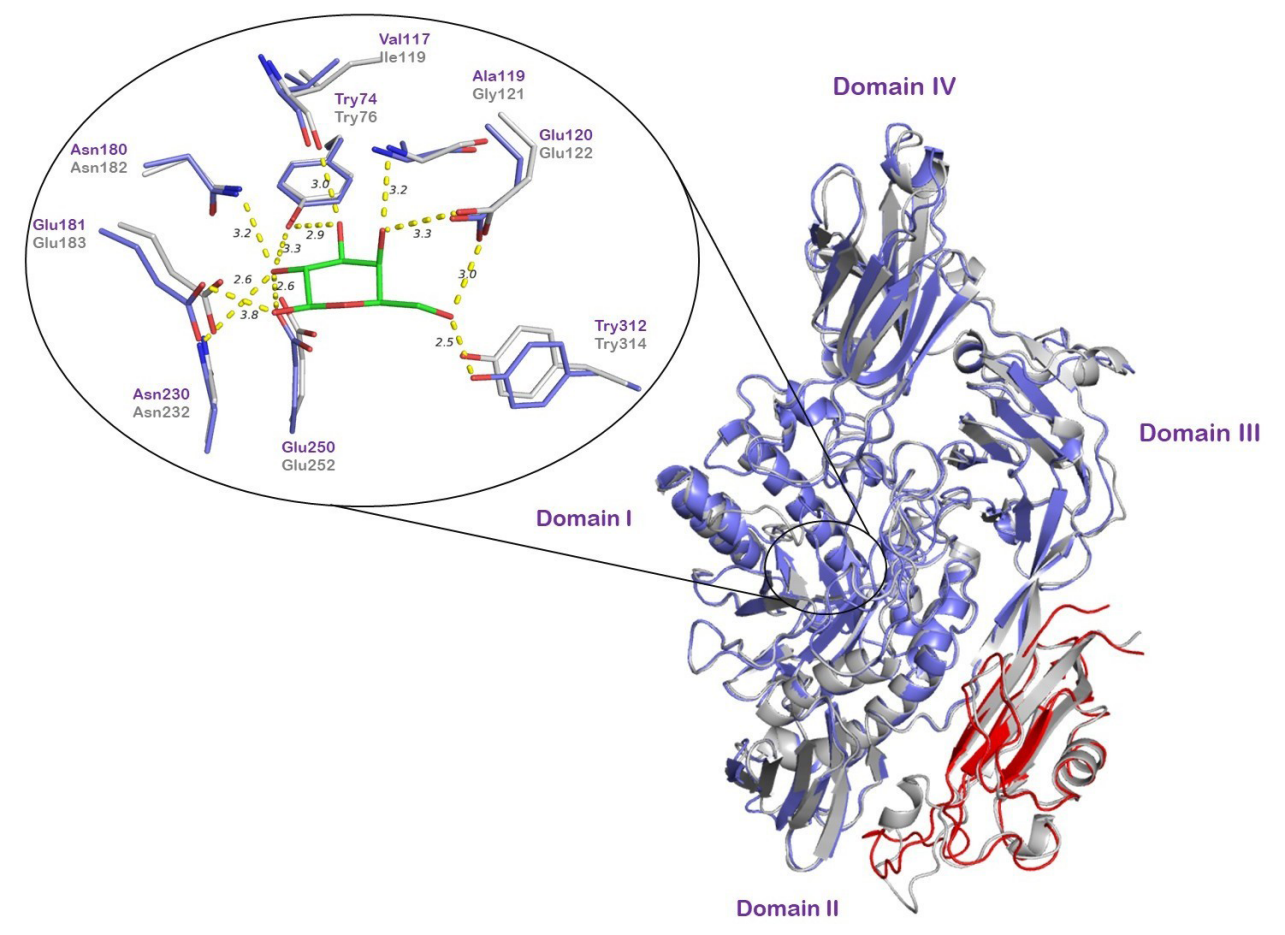
Structural superposition of the template tomato TBGal4 (purple) with the SWISS-MODEL predicted full-length model of rice *Os*BGal1(gray) and its C-terminal lectin-like domain of *Os*BGal1 (red). The superimposed active-site residues surrounding the galactose molecule from TBGal4 (green sticks). The active-site residues are highly conserved between the two enzymes, except Val117 and Ala119 in TBGal4 correspond to Ile119 and Gly121 in *O*sBGal1. Domain labels are assigned according to the crystal structure of tomato TBGal4 (Eda et al., 2016).

The *Os*BGal1 model provides a structural framework for visualizing the intact enzyme (Fig. 1), enabling comparison with tomato TBGal4, the only plant GH35 β-galactosidase with a resolved X-ray crystallographic structure (Eda et al., 2016). While the catalytic core is highly conserved, *Os*BGal1 uniquely carries a C-terminal lectin-like extension (red), which is absent from TBGal4. In the model, this domain lies against the second accessory domain of the GH35 enzyme, comprising amino acid residues S347–T412 and adopting an antiparallel β-sandwich structure composed of seven β-strands, as described in the crystal structure of tomato β-galactosidase TBGal4 (Eda et al., 2016), which interfaces with the catalytic domain, with its nearest residues ∼30 Å away from the catalytic residues in the active site (Fig. 2).

### Production of *Os*BGal1 and *Os*BGal1ΔCter

**Figure 3:**
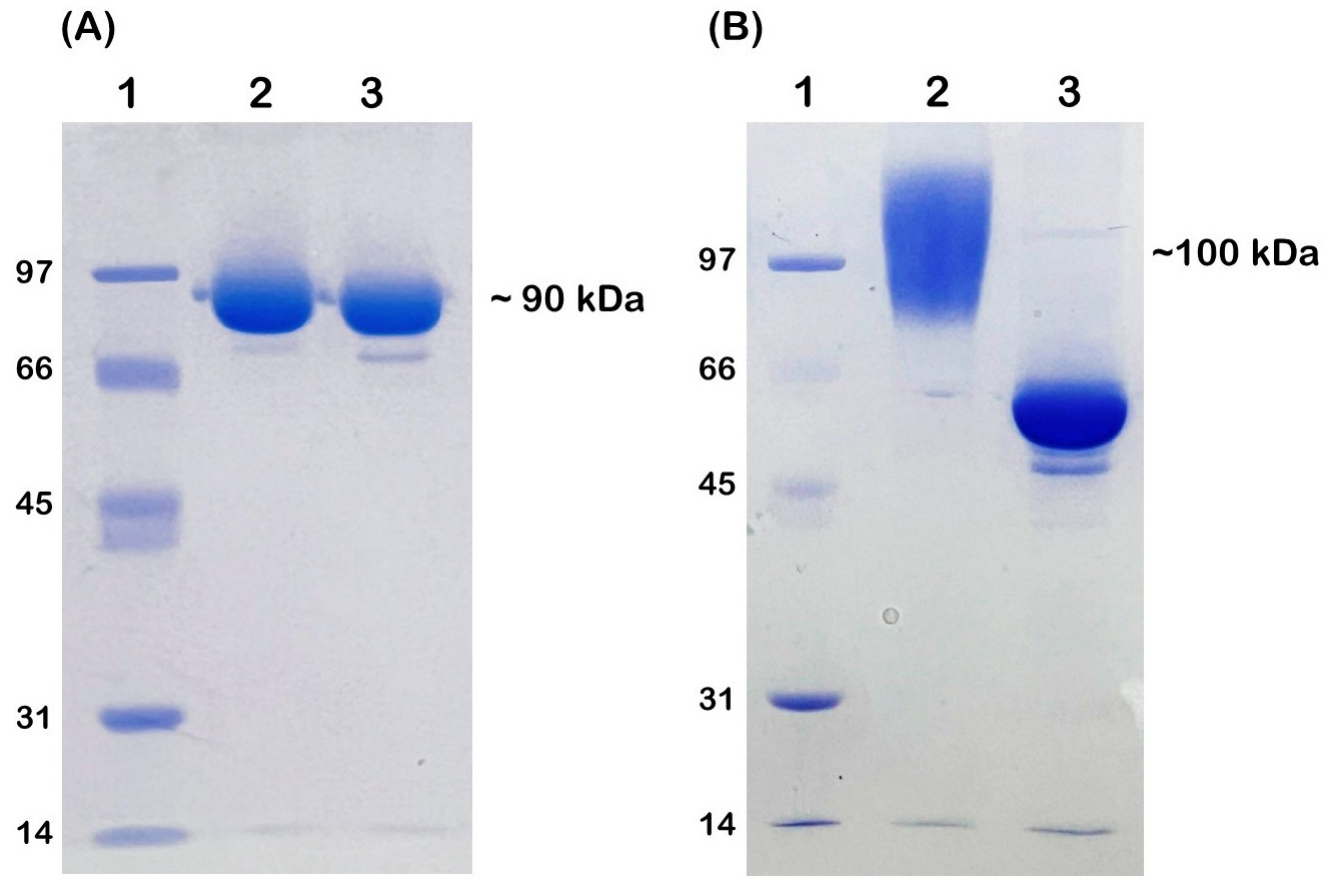
Purification of *Os*BGal1 (A) and *Os*BGal1ΔCter (B). Lane 1, protein marker; Lane 2, purified protein by IMAC, followed by S200 SEC; Lane 3, purified protein after deglycosylation with endoglycosidase H.

Recombinant *Os*BGal1 and *Os*BGal1ΔCter were successfully expressed in *P. patoris* and purified by IMAC followed by SEC (Fig. 3). The purified *Os*BGal1 migrated at an apparent molecular mass of ∼90 kDa (Fig. 3A, lane 2), whereas *Os*BGal1ΔCter appeared as a broad smear centered at >100 kDa (Fig. 3B, lane 2). After IMAC followed by SEC, *Os*BGal1 and *Os*BGal1ΔCter yielded approximately 1.2 mg and 1.5 mg of purified protein, respectively, from 1-L cultures, as shown in Table 1. Treatment with endoglycosidase H had little effect on the full-length enzyme, which migrated at a similar apparent size before and after deglycosylation, indicating a low degree of N-linked glycosylation (Fig. 3A, lane 3). In contrast, *Os*BGal1ΔCter displayed a pronounced downward mobility shift, migrating at an apparent molecular mass of ∼50–55 kDa (Fig. 3B, lane 3), which is substantially lower than its predicted molecular weight. This anomalous migration is consistent with altered SDS–PAGE behavior associated with extensive N-linked glycosylation and truncation. Following deglycosylation, *Os*BGal1ΔCter gradually lost solubility, precipitating during subsequent handling, and its enzymatic activity declined over time. *Os*BGal1 is predicted to have two glycosylation sites at Asn392 and Asn461 by the NetNGlyc program (Gupta & Brunak, 2002).

### Effect of pH and temperature on the enzyme activity of *Os*BGal1 and *Os*BGal1ΔCter

**Figure 4:**
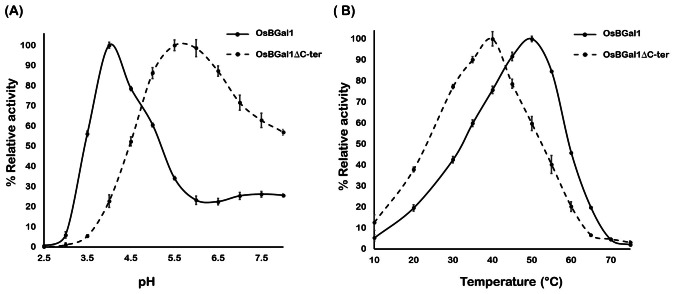
pH and temperature effects on *Os*BGal1 and *Os*BGal1ΔCter activity. (A) Activity *versus* pH curves for *Os*BGal1 and *Os*BGal1ΔCter. (B) Activity *versus* temperature curves for *Os*BGal1 and *Os*BGal1ΔCter.

The effects of pH on the enzymatic activities of *Os*BGal1 and *Os*BGal1ΔCter were determined with the *p*NP-glycoside as the substrate (Fig. 4A). *Os*BGal1 showed the highest activity at pH 4.0, with sharply decreasing activity above pH 5.0, whereas *Os*BGal1ΔCter had peak activity at approximately pH 5.0–5.5 and retained substantial residual activity across a broader pH window, maintaining more than 50% activity up to pH 8.0.

**Table 1:** Enzyme yields during purification of *Os*BGal1 and *Os*BGal1ΔCter.

**Purification step**	**Yield**		**Specific activity (units mg** ^−1^ **)**	**Purification factor (fold)**	**Recovery (%)**
	**Protein (mg)**	**Activity (units)**			
***Os*BGal1**					
Crude protein	9,182	21,589	2.4	1	100
IMAC	6	268	43	18.3	1.2
SEC	1.2	173	144	61.4	0.8
***Os*BGal1ΔCter**					
Crude protein	8,818	22,132	2.5	1	100
IMAC	6	170	29	11.4	0.8
SEC	1.5	109	74	29.3	0.5

When assayed at varied temperatures, *Os*BGal1 exhibited the highest activity at 50 °C, whereas *Os*BGal1ΔCter showed a lower optimum of 40 °C (Fig. 4B).

**Figure 5:**
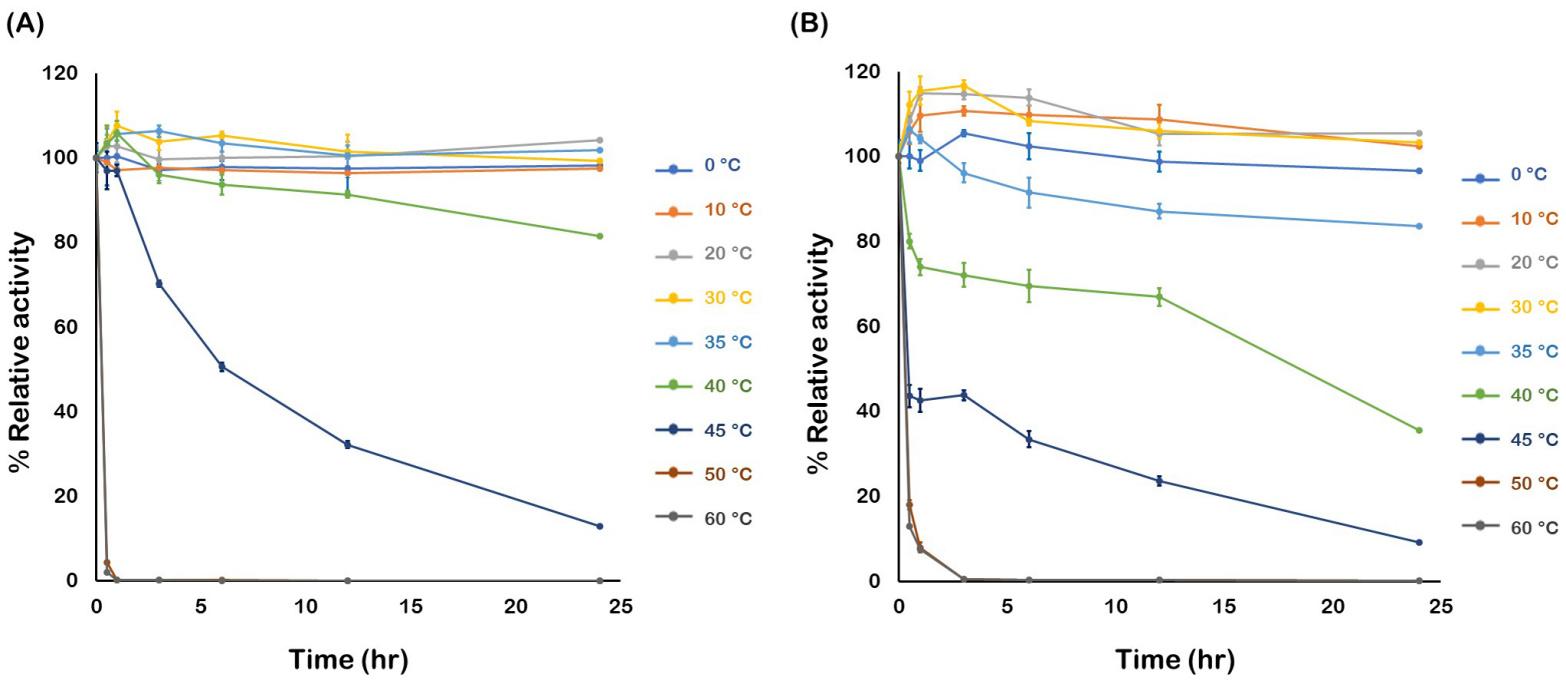
Thermal stability of *Os*BGal1 and *Os*BGal1Δ Cter. (A) Residual activity of *Os*BGal1 after incubation at different temperatures for 0–24 hr. (B) Residual activity of *Os*BGal1ΔCter under the same conditions.

The thermal stabilities of *Os*BGal1 and *Os* BGal1ΔCter were determined by monitoring residual activity after incubation at different temperatures for various time intervals. *Os*BGal1 (Fig. 5A) maintained nearly full activity at temperatures up to 40 °C for up to 24 hr, but activity declined sharply above 45 °C, with almost complete inactivation within 30 min at 50 °C and higher. In contrast, *Os*BGal1ΔCter (Fig. 5B) retained high activity only up to 30 °C and lost 25% of its activity within one hour and over 60% by 24 hr at 40 °C.

### Substrate specificity of *Os*BGal1 and *Os*BGal1ΔCter

The glycone specificity of *Os*BGal1 and *Os*BGal1ΔCter was examined with *p*NP-glycosides as substrates (Table 2). *Os*BGal1 displayed the highest activity toward *p*NPGal, with a specific activity of 133 µmol min^−1^ mg^−^^1^. Moderate activity was observed toward *p*NPFuc (22.0 µmol min^−1^ mg^−^^1^), while only low activities were detected against *p*NPGlc, *p*NPAra, and *p*NPXyl.

**Table 2:** Specific activities of *Os*BGal1 and *Os*BGal1ΔCter on *p*NP-glycosides.

**Substrate**	**Specific activity (**µmol min^−1^ mg^−1^)	
	***Os*BGal1**	***Os*BGal1ΔCter**
*p*NP-β-d- galactopyranoside	133 ± 3	18.6 ± 0.5^***^
*p*NP-β-d-glucopyranoside	1.7 ± 0.2	30.1 ± 0.5^***^
*p*NP-β-d-fucopyranoside	22.0 ± 0.5	82.9 ± 0.9^***^
*p*NP-β-d-xylopyranoside	ND	0.33 ± 0.10^*^
*p*NP-α-l-arabinopyranoside	ND	0.75 ± 0.10^**^

**Notes.**

ND not detectable

Significance values for *Os*BGal1ΔCter activities versus wildtype *Os*BGal1 were determined with the *t*-test; where wild type activity was undetectable, the test for one mean was used.

* *p* < 0.05.

** *p* < 0.01.

*** *p* < 0.0001.

*Os*BGal1ΔCter exhibited an altered activity profile. Activity toward *p*NPGal was reduced 7-fold, whereas activities toward alternative substrates were elevated (Table 2). The truncated enzyme showed specific activity toward *p*NPFuc, which was 3.8-fold higher than that of wild-type *Os*BGal1 and fourfold higher than that of *Os*BGal1ΔCter, toward *p*NPGal. Similarly, *Os*BGal1ΔCter activity toward *p*NPGlc was 17.6-fold higher than that of wild-type *Os*BGal1 and higher than its activity on *p*NPGal. *Os*BGal1ΔCter activities on *p*NPXyl and *p*NPAra were also substantially higher than those of *Os*BGal1, although lower than its activity on *p*NPGal.

### Enzyme kinetics of *Os*BGal1 and *Os*BGal1ΔCter

The kinetic parameters of *Os*BGal1 and its C-terminal truncated form were determined for the hydrolysis of *p*NPGal, *p*NPGlc, and *p*NPFuc (Table 3). For *Os*BGal1, kinetic parameters were determined for *p*NPGal and *p*NPFuc, whereas very low activity prevented the determination of these parameters for *p*NPGlc. *Os*BGal1 hydrolyzed *p*NPGal with high catalytic efficiency, characterized by a low *K*_m_ value of 0.20 mM and a *k*_cat_ of 122 s^−^^1^, resulting in a specificity constant (*k*_cat_/*K*_m_) of 596 mM^−^^1^ s^−^^1^. For *p*NPFuc, *Os*BGal1 exhibited a *K*_m_ of 28.5 mM and a *k*_cat_ of 1,077 s^−^^1^, corresponding to a lower specificity constant of 37 mM^−^^1^ s^−^^1^.

**Table 3:** Apparent kinetic parameters for *Os*BGal1 and *Os*BGal1ΔCter hydrolysis of *p*NP-glycoside substrates.

	*K*_m_ (mM)	*k*_cat_ (s^−1^)	*k*_cat_/*K*_m_ (mM^−1^ s^−1^)
***Os*BGal1**			
*p*NPGal	0.20 ± 0.01	122 ± 11	596
*p*NPGlc	ND	ND	ND
*p*NPFuc	28.5 ± 2.0	1,077 ± 2	37
***Os*BGal1ΔCter**			
*p*NPGal	3.95 ± 0.17	106 ± 0.5	27
*p*NPGlc	8.56 ± 0.46	286 ± 0.8	33
*p*NPFuc	5.78 ± 0.28	773 ± 3	133

**Notes.**

ND not detectable

Values are reported with their standard errors, based on all the data.

A clear shift in substrate specificity was observed for *Os*BGal1ΔCter. Although the enzyme retained activity on *p*NPGal, its *K*_m_ increased to 3.95 mM, although the *k*_cat_was only slightly lower at 106 s^−^^1^, yielding a specificity constant of 27 mM^−^^1^ s^−^^1^. For *p* NPGlc, the enzyme showed a *K*_m_ of 8.56 mM and a *k*_cat_of 286 s^−^^1^, corresponding to a specificity constant of 33 mM^−^^1^ s^−^^1^. For *p*NPFuc, the enzyme exhibited a *K*_m_ of 5.78 mM and a *k*_cat_ of 773 s^−^^1^, resulting in a specificity constant of 133 mM^−^^1^ s^−^^1^.

### Transglycosylation activity of *Os*BGal1 and *Os*BGal1ΔCter

**Figure 6:**
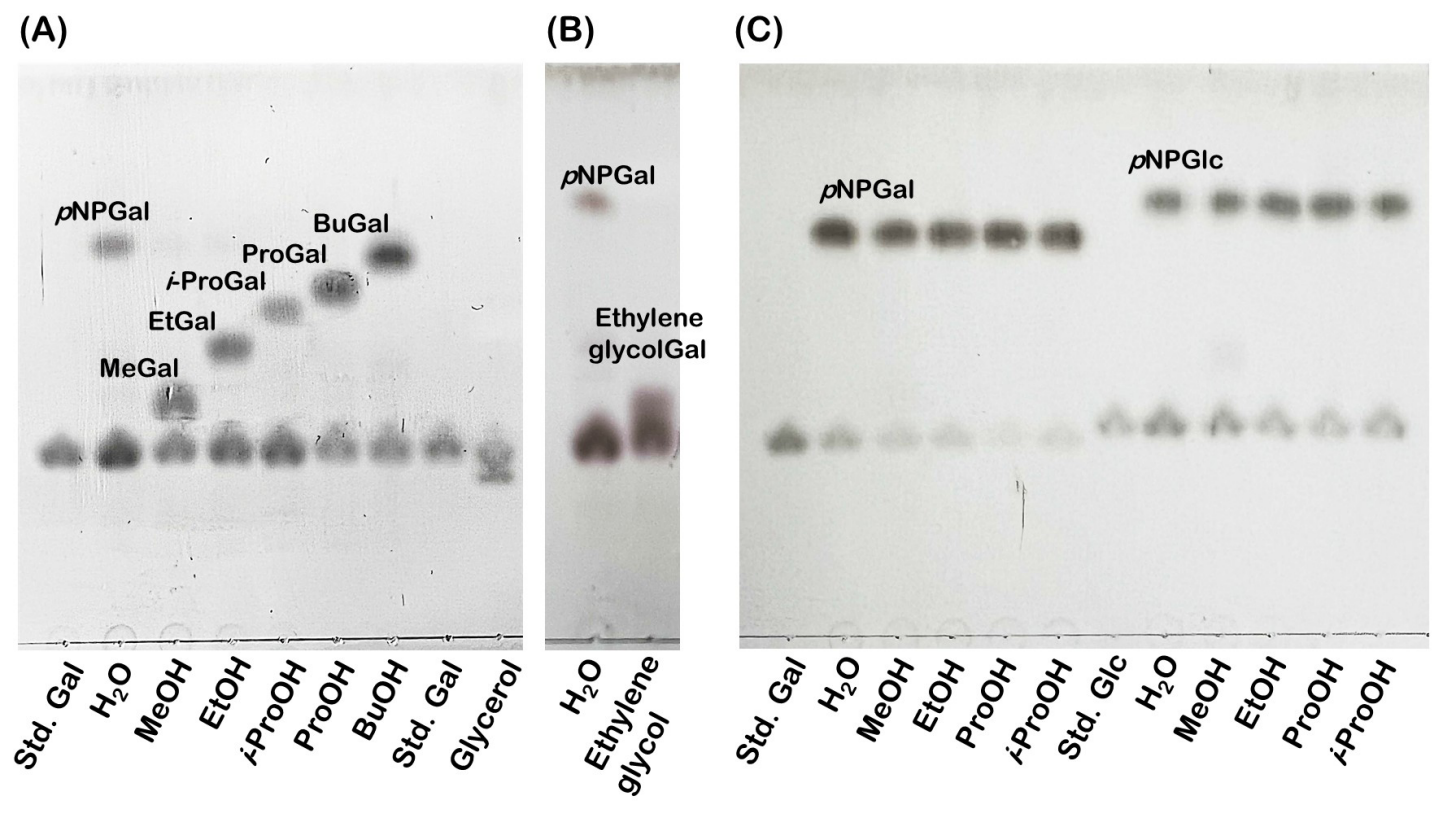
Transglycosylation products of *Os*BGal1 and the *Os*BGal1ΔCter analyzed by TLC. (A) *Os*BGal1 transglycosylation using *p*NPGal as a donor with methanol (MeOH), ethanol (EtOH), *n*-propanol (ProOH), isopropanol (*i*ProOH), butanol (BuOH) and glycerol as acceptors. Distinct transglycosylation products (MeGal, EtGal, ProGal, *i*ProGal, BuGal and glycerol-Gal) are observed. (B) *Os*BGal1 transglycosylation using *p*NPGal as a donor with ethylene glycol as acceptors. Ethylene glycol supports the formation of clear products (ethylene glycol–Gal). This reaction was first developed in a chloroform:methanol (4:1, v/v) mixture, followed by a butanol:acetic acid:water (2:1:1, v/v/v) mixture for optimal resolution. (C) *Os*BGal1ΔCter tested with *p*NPGal and *p*NPGlc as donors and methanol, ethanol, *n*-propanol, and isopropanol as acceptors. Transglycosylation products are absent or faint compared to wild-type *Os*BGal1.

To investigate the transglycosylation activity of *Os*BGal1 and *Os*BGal1ΔCter, *p*NPGal and *p*NPGlc were tested as sugar donors, and several alcohols were examined as acceptors, including methanol, ethanol, *n*-propanol, isopropanol, butanol, glycerol, and ethylene glycol. TLC analysis of the *Os*BGal1 reaction products revealed clear formation of new glycoside spots in the presence of acceptors, corresponding to methyl galactoside (MeGal), ethyl galactoside (EtGal), propyl galactoside (ProGal), isopropyl galactoside (*i*-ProGal), butyl galactoside (BuGal), and glycerol galactoside (glycerolGal) were observed (Fig. 6A). Ethylene glycol also served as an efficient acceptor to produce its galactoside (ethylene glycolGal) (Fig. 6B).

In contrast, the *Os*BGal1ΔCter displayed very little transglycosylation activity (Fig. 6C). *Os* BGal1ΔCter was tested with *p*NPGal and *p*NPGlc as donors in the presence of methanol, ethanol, *n*-propanol, and isopropanol. With both donors, only very weak product bands were detected in the methanol reaction, while no detectable transglycosylation products were observed with other acceptors.

### Molecular dynamics simulation

**Figure 7:**
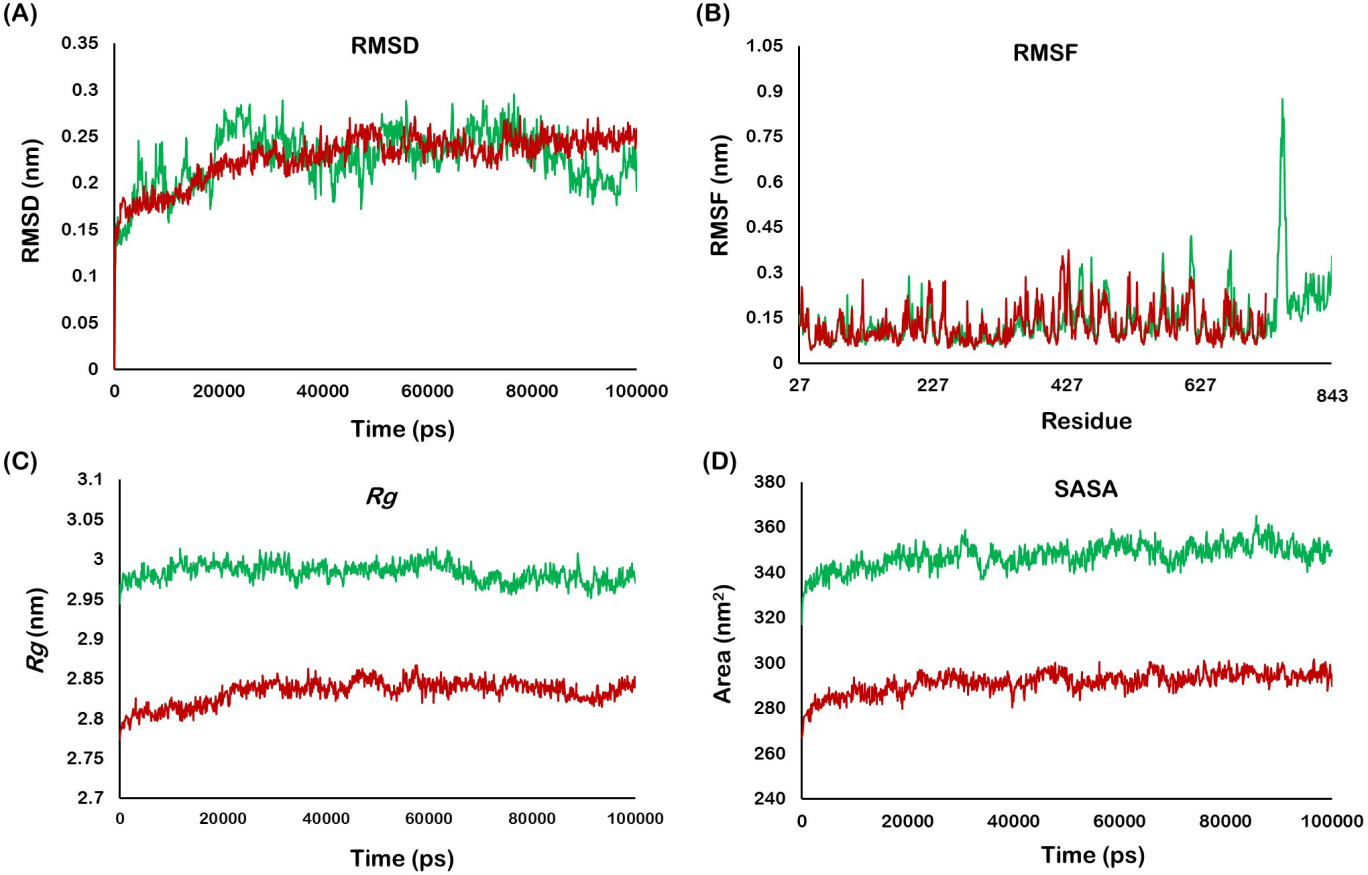
MD analysis of *Os*BGal1 (green) and *Os*BGal1ΔCter (maroon) over 100 ns. (A) The *Os*BGal1 stabilized at 0.25 nm, while *Os*BGal1ΔCter maintained a similar RMSD but with slightly lower amplitude. (B) RMSF per residue fluctuated below 0.25 nm; a distinct flexibility peak around residues 739–760 was observed in loop A, located within the C-terminal lectin-like domain. (C) The *Rg* of *Os*BGal1 was slightly larger than that of *Os*BGal1ΔCter by 0.15 nm, and both traces are flat, confirming structural compactness. (D) Total SASA of *Os*BGal1 was greater than that of *Os*BGal1ΔCter by 40–55 nm^2^, consistent with the presence of the extra domain.

The structural dynamics of *Os*BGal1 and *Os*BGal1ΔCter were examined over 100 ns of molecular dynamics simulations, which showed that both constructs remained folded and compact throughout (Fig. 7). The root-mean-square deviation (RMSD) in both systems equilibrated within the first ∼20 ns. The *Os*BGal1 (green) stabilized at ∼0.25 nm, while *Os*BGal1ΔCter (maroon) maintained a similar RMSD (∼0.24 nm), indicating comparable backbone stability and the absence of significant conformational drift (Fig. 7A). Per-residue root mean square fluctuation (RMSF) profiles were broadly similar. Most residues fluctuated below 0.25 nm, indicating a rigid backbone in both enzymes. A distinct flexibility peak around residues 739–760 was observed in loop A, located within the C-terminal lectin-like domain, which is absent in the truncated enzyme (Fig. 7B). The radius of gyration (*Rg*) indicating global compactness was steady and flat over time for both systems (Fig. 7C). The *Os*BGal1 maintained a larger radius (∼2.99 nm) than *Os*BGal1ΔCter (∼2.84 nm), a difference of ∼0.15 nm consistent with the added domain, with no evidence of unfolding in either system. Total solvent-accessible surface area (SASA) was correspondingly higher for *Os*BGal1 (∼350–365 ± 10 nm^2^) than for *Os*BGal1ΔCter (∼290–310 nm^2^) (Fig. 7D), as expected for a larger protein.

## Discussion

### Glycosylation of *Os*BGal1 and *Os*BGal1ΔCter

**Figure 8:**
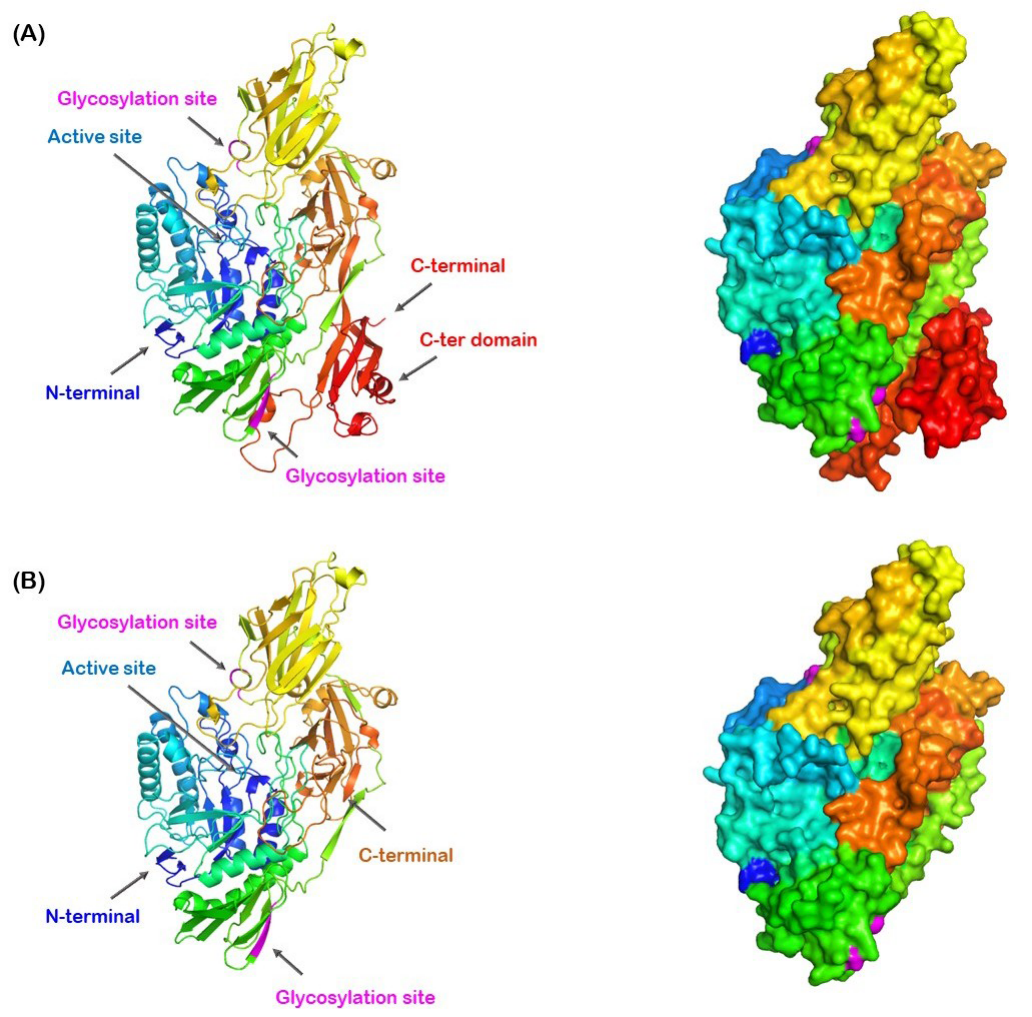
Comparison of the *Os*BGal1 and *Os*BGal1ΔCter structural models predicted by SWISS-MODEL. (A) Predicted structure of *Os*BGal1. (B) Predicted structure of *Os*BGal1ΔCter, in which the C-terminal domain is absent. The models are colored from blue (N-terminus) to red (C-terminus). Glycosylation sites (magenta) and the predicted active site (blue) are indicated. Ribbon diagrams are shown on the left, and surface representations on the right. Coloring follows the same spectrum from N- to C-terminus.

*Os*BGal1 was expressed in *P. pastoris* to enable glycosylation, but this also led to differences in glycosylation between the full-length and truncated proteins. The difference in apparent SDS-PAGE patterns between *Os*BGal1 and *Os*BGal1ΔCter can be attributed to altered glycosylation. The effect of Endo H digestion on *Os*BGal1 suggests that glycosylation is limited in the full-length enzyme (Fig. 3A). In contrast, the distinct Endo H sensitivity of *Os*BGal1ΔCter indicates that removal of the C-terminal lectin-like domain exposes the nearby glycosylation site, resulting in hyperglycosylation (Fig. 3B), although the protein had an apparent molecular weight of approx. 55 kDa, suggesting it may have been degraded during Endo H digestion. Structural prediction of *Os*BGal1 by SWISS-MODEL placed the potential N-glycosylation site at Asn392 near the C-terminal lectin-like domain (Fig. 8). In the full-length model, this site appeared partially shielded by the C-terminal domain. In contrast, the *Os*BGal1ΔCter model lacked the C-terminal domain, leaving Asn392 fully exposed on the protein surface. This suggests that the C-terminal lectin-like domain of *Os*BGal1 may block access of the glycosylation machinery to Asn392. This structural difference is consistent with the higher apparent glycosylation of *Os*BGal1ΔCter observed by SDS–PAGE (Fig. 3).

The gradual precipitation and progressive loss of activity observed in Endo H-treated *Os*BGal1ΔCter suggest that glycosylation contributes significantly to the stability and/or solubility of the truncated enzyme (Kwon & Yu, 1997). In the absence of the C-terminal lectin-like domain, glycans may compensate for reduced structural stability or solubility (Shental-Bechor & Levy, 2009). Once removed, the protein becomes increasingly unstable, eventually precipitating and losing activity. This contrasts with the full-length enzyme, where the C-terminal domain likely shields glycosylation sites and supports stability independent of extensive glycan modification (Bretthauer & Castellino, 1999).

On the other hand, the hyperglycosylation caused by the removal of the C-terminal domain could cause some of the effects on catalysis. The large high-mannose glycan of yeast N-glycosylation is different from the smaller, complex glycans found in plants, with only 3 mannose residues substituted with N-acetylglucosamine and xylose, along with fucose on the initial N-acetylglucosamine (Strasser, 2016). Moreover, as noted above, glycosylation of Asn392 may be blocked in the full-length protein, resulting in extensive glycosylation at an unusual site in the truncation. Since the glycosylation sites are on surfaces distant from the active site and are pointed away from it, any effects would likely be indirect. Still, they cannot be ruled out as a cause of the observed change in catalytic behavior.

### Effect of pH and temperature on the enzyme activity of *Os*BGal1 and *Os*BGal1ΔCter

The comparison between *Os*BGal1 and its truncated form *Os*BGal1ΔCter emphasizes the role of the C-terminal lectin-like domain in shaping the catalytic environment of the enzyme. The full-length enzyme exhibits a sharp activity optimum under acidic pH and elevated temperature, consistent with the physiological conditions of the plant cell wall, where plant GH35 β-galactosidases are expected to operate during cell wall loosening and remodeling (Smith & Gross, 2000; Paniagua et al., 2016). In contrast, deletion of the C-terminal domain broadens the pH preference toward a neutral range, increasing the pH optimum by two pH units. This suggests that the C-terminal domain somehow modulates the electrostatic environment of the active site. Since the C-terminal domain contains an equal number of positively and negatively charged residues (10 Arg/Lys and 10 Asp/Glu, pI 7.0), it is unlikely to be due to a long-range electrostatic effect. Removal of the C-terminal domain decreased the temperature optimum, indicating that it contributes to the enzyme’s stability. The full-length enzyme retained activity longer at 40–45 °C, whereas *Os*BGal1ΔCter was rapidly inactivated under the same conditions. These findings imply that the C-terminal domain not only supports optimal catalysis under the acidic microenvironments of the plant apoplast, where it is thought to be located (Chantarangsee et al., 2007), but also enhances enzyme robustness. Its removal results in a less specialized enzyme that retains activity over a broader pH range but at the expense of efficiency and thermostability. This observation supports the function of the C-terminal domain in stabilizing the catalytic core against heat-induced denaturation and in maintaining the enzyme’s functional conformation during prolonged incubation (Petrova et al., 2021).

### Substrate specificity of *Os*BGal1 and *Os*BGal1ΔCter

The C-terminal lectin-like domain of *Os*BGal1 contributes to substrate specificity despite being quite distant from the active site in the structural model. The full-length enzyme exhibited high activity toward *p*NPGal, consistent with the substrate preferences reported for plant GH35 β-galactosidases (Ahn et al., 2007; Eda et al., 2016; Chantarangsee et al., 2007; Trainotti et al., 2001), with minimal activity toward other glycosides, except for β-d-fucoside. In contrast, *Os*BGal1ΔCter behaved quite differently. Its activity on *p*NPGal was much lower, and its specificity constant was nearly 20-fold lower, reflecting a corresponding 20-fold increase in *K*_m_. In contrast, it showed significantly higher activity on glucoside, fucoside, and xyloside, with the highest specificity for *p*NP-β-d-fucoside, followed by *p*NP-β-d-glucoside. This change means that the C-terminal domain is crucial for maintaining the enzyme’s specificity for galactosides while limiting its activity on other sugars (Chandrasekar & Ra, 2016). These changes suggest that the C-terminal domain is not essential for catalysis itself, as *k*_cat_ values remained comparable or even higher; however, it is critical for maintaining substrate selectivity and stabilizing galactoside binding. The absence of the C-terminal domain or the extensive glycosylation in *Os*BGal1ΔCter may expose or alter the substrate-binding environment, thereby reducing the constraints that favor galactose recognition while allowing alternative substrates to be hydrolyzed. Selection for β-galactosides generally relies on two components: selection for the axial 4-hydroxyl group (as opposed to the equatorial 4-hydroxyl in glucose and xylose) and positioning of the side chain in a less activating position to allow the more active galactoside to react but not less active sugars like glucose (Quirke & Crich, 2020). Thus, some structural change in the interactions with the C4 hydroxyl or the C6H_2_OH side chain of the sugar should be necessary to change the glycone specificity. This suggests that either long-range interactions are conveyed through the protein structure to the active site, or the structural model has misplaced at least part of the C-terminal domain, which should be in proximity to the active site in order to change the positions of interacting groups within the glycone-binding site. Notably, both AlphaFold 3 and SWISS-MODEL predicted a similar position for the C-terminal domain.

Both GH35 enzymes, with and without the C-terminal domain, have similar substrate specificities, so enzymes lacking the domain have evolved other mechanisms to maintain β-galactoside specificity. This specificity is important, since their roles in plant cell wall remodeling require them to remove only galactose. For instance, *At*BGAL6 removes only galactosyl side chains from rhamnogalacturonan I, thereby allowing muscilage extrusion (Macquet et al., 2007). If it were to remove other types of side chains, the properties of the mucilage may be affected.

### Transglycosylation activity of *Os*BGal1 and *Os*BGal1ΔCter

Our results show that *Os*BGal1 requires its C-terminal lectin-like domain to catalyze transglycosylation, whereas the *Os*BGal1ΔCter retained hydrolytic activity but lost nearly all sugar transfer capacity. This finding highlights a domain-dependent mechanism that distinguishes *Os*BGal1 from other plant GH35 enzymes. For example, tomato TBGal4 β-galactosidase and rice *Os*BGal2, both of which lack the C-terminal domain, display substrate specificities similar to lectin-like-domain-containing β-galactosidases (Chantarangsee et al., 2007; Eda et al., 2016). Thus, the lectin-like extension is not universally required for catalysis within GH35. The role of the C-terminal domain may be context-dependent. In *Os*BGal1, it may help position acceptor substrates and limit solvent access, creating a microenvironment favorable for glycosidic bond formation. Alternatively, the extensive glycosylation seen in the truncated protein may disrupt this microenvironment. Because transglycosylation requires precise placement of the acceptor and restricted access to water, the full-length enzyme could promote sugar transfer by shielding the active site. Without this domain, the catalytic pocket is likely more solvent-exposed, favoring hydrolysis and permitting broader substrate binding. Thus, the lectin-like domain cloud appears to act as both a specificity filter and a structural shield, enabling efficient hydrolysis and transglycosylation, whereas its absence yields a more flexible but less specialized enzyme.

### Molecular dynamics simulation

Across the simulated timescales, both *Os*BGal1 and *Os*BGal1ΔCter remained folded and compact. *Os*BGal1 exhibited slightly higher global RMSD, along with larger SASA and *Rg*, attributed to its expanded architecture resulting from the C-terminal lectin-like domain rather than instability of the catalytic barrel. The *Os*BGal1ΔCter was overall more compact, with lower SASA and *Rg*. RMSF profiles were broadly similar, consistent with stable cores in both proteins. Taken together, the MD simulations suggest that the full-length and truncated structures predicted by SWISS-MODEL are reasonable and stable, but they cannot readily account for the functional differences between the full-length and truncated forms of *Os*BGal1. The C-terminal domain may dampen and organize inter-domain motions, while leaving the catalytic barrel intact. Its catalytic influence is probably indirect, unless structure prediction misplaces the C-terminal domain and its actual location is near the mouth of the active site.

### General discussion

The discovery that removing the C-terminal domain could alter the enzyme’s glycone specificity was unexpected, as it was expected to play an auxiliary role, such as helping to bind to cell wall substrates. Thus, the change in activity on small substrates that cannot extend out of the active site during hydrolysis was not anticipated. Initially, we planned to produce the enzyme with and without this domain for structural studies by X-ray crystallography, small-angle X-ray scattering (SAX), or cryogenic electron microscopy (Cryo-EM). It was expected that the enzyme might maintain its basic function, but placement of the C-terminal domain would allow us to better understand the functional implications of this domain. Although we were thus far unable to determine the structure by any of these means, we instead found a remarkable functional shift in glycone specificity and transglycosylation ability. Although we might have hypothesized a change in activity on large, complex natural substrates such as galactans and arabinogalactans, which could have extensive binding across the protein surface, the change in activity on small synthetic glycosides was surprising.

The unexpected functional shift opens the possibility of engineering *Os*BGal1 to meet the needs of applications requiring broader substrate specificity. The *Os*BGalΔCter can act on additional substrates. It is possible that, instead of removal, the domain sequence could be modified to improve some properties while retaining others. Furthermore, the *Os*BGalΔCter could be engineered to improve stability and other properties, making it more desirable for industrial applications.

## Conclusions

The C-terminal domain of rice GH35 β-galactosidases1 plays a key role in determining both substrate selectivity and catalytic function. Comparison between full-length and C-terminal truncated enzymes revealed that removal of this domain significantly reduces catalytic efficiency, indicating its importance in stabilizing substrate binding and facilitating turnover. In addition, the truncated enzyme loses preference for **β-**d-galactoside, demonstrating that the C-terminal domain contributes to selective recognition of galactoside substrates, even though plant GH35 enzymes without this domain, like TBGal4, have similar specificities as full-length *Os*BGal1. Furthermore, the transglycosylation activity was decreased in the absence of the C-terminal domain, suggesting its involvement in proper substrate positioning for both hydrolysis and transglycosylation reactions. Evidently, either *Os*BGal1 has evolved to rely on the C-terminal domain for its activity, or the hyperglycosylation that occurs upon its removal affects this activity. Our findings not only clarify the effect of the C-terminal domain on plant GH35 enzymes but also suggest that its removal could alter specificity, enabling the use of this deletion strategy in glycoengineering and plant biotechnology.

##  Supplemental Information

10.7717/peerj.21066/supp-1 Supplemental Information 1 *Os* BGal1 and *Os* BGal1ΔCter genes codon-optimized for expression in *Pichia pastoris*

10.7717/peerj.21066/supp-2 Supplemental Information 2 The raw measurement data for enzyme characterization and kinetics

10.7717/peerj.21066/supp-3 Supplemental Information 3 Trajectory of the 100 ns molecular dynamics simulation of *Os* BGal1 The stability and compact structure of the full-length Os BGal1 during the 100 ns molecular dynamics simulation.

10.7717/peerj.21066/supp-4 Supplemental Information 4 Trajectory of the 100 ns molecular dynamics simulation of *Os* BGal1 ΔCter The stability and compact structure of *Os* BGal1ΔCter without the C-terminal lectin-like domain during the 100 ns molecular dynamics simulation.

## Additional Information and Declarations

### DNA Deposition

The following information was supplied regarding the deposition of DNA sequences: The sequences are available at DDBJ: LC898194.

### Data Availability

The following information was supplied regarding data availability: The raw data is available in the Supplemental Files. The molecular dynamics input files and PDB files are available at FigShare: - Choknud, Sunaree; Sanram, Surapoj; R. Ketudat Cairns, James (2025). Molecular dynamics simulation data for GH35 rice β-galactosidases1. figshare. Dataset. https://doi.org/10.6084/m9.figshare.30520754.v1
The sequences are available at DDBJ: LC898194.
