# Supplementary material for: The C-terminal lectin-like domain modulates the substrate specificity and transglycosylation activity of rice β-galactosidase1 (OsBGal1)
Source: PeerJ. 2026 Apr 9;14:e21066. doi: 10.7717/peerj.21066 (PMC13070324; doi:10.7717/peerj.21066)
Supplement: Supplemental Information 1 [file peerj-14-21066-s001.docx]

**The C-terminal lectin-like domain modulates the substrate specificity and transglycosylation activity of rice β-galactosidase1 (OsBGal1)**

Sunaree Choknud^1, 2^, Thipwarin Rimlumduan^3^, Surapoj Sanram^4^, Jenis Chiawitthayanan^1, 2^, Wipa Suginta^4^, James R. Ketudat Cairns^1, 2^

^1^School of Chemistry, Institute of Science, Suranaree University of Technology, Nakhon Ratchasima, 30000 Thailand

^2^ Center for Biomolecular Structure, Function and Application, Suranaree University of Technology, Nakhon Ratchasima, 30000 Thailand

^3^ Department of Applied Biology, Faculty of Science and Liberal Arts, Rajamangala University of Technology Isan, Nakhon Ratchasima, 30000 Thailand

^4^ School of Biomolecular Science and Engineering, Vidyasirimedhi Institute of Science and Technology Rayong 21210, Thailand

Corresponding Author:

James R. Ketudat Cairns^1,2^

111 University Avenue Muang Nakhon Ratchasima, 30000, Thailand

Email address: [cairns@sut.ac.th](mailto:cairns@sut.ac.th)

**Contents:**

| Description | Page |
| --- | --- |
| *Os*BGal1 and *Os*BGal1∆Cter genes codon-optimized for expression in *Pichia pastoris*. | 2 |
| Supplementary Figure 1. Schematic representation of the expression constructs used in this study. | 7 |

***Os*BGal1 and *Os*BGal1∆Cter genes codon-optimized for expression in *Pichia pastoris*.**

The optimized nucleotide sequence (lowercase letters) and the corresponding amino-acid translation (uppercase letters) of the *Os*BGal1 and *Os*BGal1∆Cter genes that were codon-optimized for expression in *Pichia pastoris*. The truncated nucleotide and protein sequences are shown in grey highlight. Stop codons are represented by hyphens (-).

gtt act tac gat aag aaa gct gtt ttg gtt gac ggt caa aga aga att ttg ttt tcc gga

gtt act tac gat aag aaa gct gtt ttg gtt gac ggt caa aga aga att ttg ttt tcc gga

V T Y D K K A V L V D G Q R R I L F S G

V T Y D K K A V L V D G Q R R I L F S G

tca atc cat tat cct aga tct aca cca gaa atg tgg gat ggt ttg att gag aag gct aaa

tca atc cat tat cct aga tct aca cca gaa atg tgg gat ggt ttg att gag aag gct aaa

S I H Y P R S T P E M W D G L I E K A K

S I H Y P R S T P E M W D G L I E K A K

gat ggt gga ttg gac gtt atc caa act tac gtt ttc tgg aat gga cat gaa cca aca cct

gat ggt gga ttg gac gtt atc caa act tac gtt ttc tgg aat gga cat gaa cca aca cct

D G G L D V I Q T Y V F W N G H E P T P

D G G L D V I Q T Y V F W N G H E P T P

ggt aac tac aat ttc gag gga aga tac gat ttg gtt aga ttc att aag act gtt caa aaa

ggt aac tac aat ttc gag gga aga tac gat ttg gtt aga ttc att aag act gtt caa aaa

G N Y N F E G R Y D L V R F I K T V Q K

G N Y N F E G R Y D L V R F I K T V Q K

gct gga atg ttt gtt cac ttg aga att gga cca tac atc tgt ggt gaa tgg aac ttt ggt

gct gga atg ttt gtt cac ttg aga att gga cca tac atc tgt ggt gaa tgg aac ttt ggt

A G M F V H L R I G P Y I C G E W N F G

A G M F V H L R I G P Y I C G E W N F G

gga ttc cct gtt tgg ttg aag tat gtt cca ggt att tcc ttt aga act gat aat gag cct

gga ttc cct gtt tgg ttg aag tat gtt cca ggt att tcc ttt aga act gat aat gag cct

G F P V W L K Y V P G I S F R T D N E P

G F P V W L K Y V P G I S F R T D N E P

ttc aaa aac gct atg caa ggt ttt aca gaa aag atc gtt gga atg atg aaa tca gag aat

ttc aaa aac gct atg caa ggt ttt aca gaa aag atc gtt gga atg atg aaa tca gag aat

F K N A M Q G F T E K I V G M M K S E N

F K N A M Q G F T E K I V G M M K S E N

ttg ttc gct tct caa ggt gga cct att atc ttg tct cag att gaa aac gag tac ggt cca

ttg ttc gct tct caa ggt gga cct att atc ttg tct cag att gaa aac gag tac ggt cca

L F A S Q G G P I I L S Q I E N E Y G P

L F A S Q G G P I I L S Q I E N E Y G P

gaa gga aag gag ttt ggt gct gct gga aaa gct tat atc aat tgg gct gct aag atg gct

gaa gga aag gag ttt ggt gct gct gga aaa gct tat atc aat tgg gct gct aag atg gct

E G K E F G A A G K A Y I N W A A K M A

E G K E F G A A G K A Y I N W A A K M A

gtt ggt ttg gat act gga gtt cca tgg gtt atg tgt aaa gaa gat gac gct cca gat cct

gtt ggt ttg gat act gga gtt cca tgg gtt atg tgt aaa gaa gat gac gct cca gat cct

V G L D T G V P W V M C K E D D A P D P

V G L D T G V P W V M C K E D D A P D P

gtt att aat gct tgt aac ggt ttt tac tgt gac act ttc tca cca aac aag cct tat aaa

gtt att aat gct tgt aac ggt ttt tac tgt gac act ttc tca cca aac aag cct tat aaa

V I N A C N G F Y C D T F S P N K P Y K

V I N A C N G F Y C D T F S P N K P Y K

cca act atg tgg aca gaa gct tgg tct gga tgg ttt act gag ttc ggt gga aca atc aga

cca act atg tgg aca gaa gct tgg tct gga tgg ttt act gag ttc ggt gga aca atc aga

P T M W T E A W S G W F T E F G G T I R

P T M W T E A W S G W F T E F G G T I R

caa aga cca gtt gaa gat ttg gct ttt ggt gtt gct aga ttc gtt cag aag ggt gga tct

caa aga cca gtt gaa gat ttg gct ttt ggt gtt gct aga ttc gtt cag aag ggt gga tct

Q R P V E D L A F G V A R F V Q K G G S

Q R P V E D L A F G V A R F V Q K G G S

ttt att aac tac tac atg tac cat ggt gga act aac ttc ggt aga aca gct ggt gga cct

ttt att aac tac tac atg tac cat ggt gga act aac ttc ggt aga aca gct ggt gga cct

F I N Y Y M Y H G G T N F G R T A G G P

F I N Y Y M Y H G G T N F G R T A G G P

ttt atc act aca tcc tac gat tat gac gct cct ttg gat gaa tat ggt ttg gct aga gag

ttt atc act aca tcc tac gat tat gac gct cct ttg gat gaa tat ggt ttg gct aga gag

F I T T S Y D Y D A P L D E Y G L A R E

F I T T S Y D Y D A P L D E Y G L A R E

cca aag ttc gga cat ttg aaa gaa ttg cac aga gct gtt aaa ttg tgt gag caa cct ttg

cca aag ttc gga cat ttg aaa gaa ttg cac aga gct gtt aaa ttg tgt gag caa cct ttg

P K F G H L K E L H R A V K L C E Q P L

P K F G H L K E L H R A V K L C E Q P L

gtt tct gct gac cca act gtt act aca ttg gga tcc atg cag gaa gct cac gtt ttc aga

gtt tct gct gac cca act gtt act aca ttg gga tcc atg cag gaa gct cac gtt ttc aga

V S A D P T V T T L G S M Q E A H V F R

V S A D P T V T T L G S M Q E A H V F R

tct tcc tca ggt tgt gct gct ttc ttg gct aac tac aat tct aac tcc tac gct aag gtt

tct tcc tca ggt tgt gct gct ttc ttg gct aac tac aat tct aac tcc tac gct aag gtt

S S S G C A A F L A N Y N S N S Y A K V

S S S G C A A F L A N Y N S N S Y A K V

att ttc aac aac gag aac tac tct ttg cca cct tgg tca atc tct atc ttg cca gat tgt

att ttc aac aac gag aac tac tct ttg cca cct tgg tca atc tct atc ttg cca gat tgt

I F N N E N Y S L P P W S I S I L P D C

I F N N E N Y S L P P W S I S I L P D C

aag aac gtt gtt ttt aac act gct aca gtt ggt gtt caa act aac caa atg cag atg tgg

aag aac gtt gtt ttt aac act gct aca gtt ggt gtt caa act aac caa atg cag atg tgg

K N V V F N T A T V G V Q T N Q M Q M W

K N V V F N T A T V G V Q T N Q M Q M W

gct gac gga gct tct tcc atg atg tgg gaa aag tat gat gaa gag gtt gac tca ttg gct

gct gac gga gct tct tcc atg atg tgg gaa aag tat gat gaa gag gtt gac tca ttg gct

A D G A S S M M W E K Y D E E V D S L A

A D G A S S M M W E K Y D E E V D S L A

gct gct cca ttg ttg act tct aca ggt ttg ttg gag caa ttg aac gtt act aga gat aca

gct gct cca ttg ttg act tct aca ggt ttg ttg gag caa ttg aac gtt act aga gat aca

A A P L L T S T G L L E Q L N V T R D T

A A P L L T S T G L L E Q L N V T R D T

tct gac tac ttg tgg tat att act aga gtt gaa gtt gat cct tcc gag aaa ttc ttg caa

tct gac tac ttg tgg tat att act aga gtt gaa gtt gat cct tcc gag aaa ttc ttg caa

S D Y L W Y I T R V E V D P S E K F L Q

S D Y L W Y I T R V E V D P S E K F L Q

ggt gga act cca ttg tct ttg aca gtt cag tcc gct ggt cat gct ttg cac gtt ttt att

ggt gga act cca ttg tct ttg aca gtt cag tcc gct ggt cat gct ttg cac gtt ttt att

G G T P L S L T V Q S A G H A L H V F I

G G T P L S L T V Q S A G H A L H V F I

aat ggt caa ttg cag gga tca gct tac ggt act aga gaa gac aga aag atc tcc tat tct

aat ggt caa ttg cag gga tca gct tac ggt act aga gaa gac aga aag atc tcc tat tct

N G Q L Q G S A Y G T R E D R K I S Y S

N G Q L Q G S A Y G T R E D R K I S Y S

ggt aat gct aac ttg aga gct gga aca aac aaa gtt gct ttg ttg tct gtt gct tgt gga

ggt aat gct aac ttg aga gct gga aca aac aaa gtt gct ttg ttg tct gtt gct tgt gga

G N A N L R A G T N K V A L L S V A C G

G N A N L R A G T N K V A L L S V A C G

ttg cca aat gtt ggt gtt cat tac gaa act tgg aac aca ggt gtt gtt gga cct gtt gtt

ttg cca aat gtt ggt gtt cat tac gaa act tgg aac aca ggt gtt gtt gga cct gtt gtt

L P N V G V H Y E T W N T G V V G P V V

L P N V G V H Y E T W N T G V V G P V V

att cac gga ttg gat gag ggt tcc aga gac ttg act tgg caa aca tgg tca tat caa ttt

att cac gga ttg gat gag ggt tcc aga gac ttg act tgg caa aca tgg tca tat caa ttt

I H G L D E G S R D L T W Q T W S Y Q F

I H G L D E G S R D L T W Q T W S Y Q F

cag gtt ggt ttg aag gga gaa cag atg aat ttg aac tcc ttg gaa ggt tct gga tcc gtt

cag gtt ggt ttg aag gga gaa cag atg aat ttg aac tcc ttg gaa ggt tct gga tcc gtt

Q V G L K G E Q M N L N S L E G S G S V

Q V G L K G E Q M N L N S L E G S G S V

gag tgg atg caa ggt tca ttg gtt gct cag aat caa cag cca ttg gct tgg tac aga gct

gag tgg atg caa ggt tca ttg gtt gct cag aat caa cag cca ttg gct tgg tac aga gct

E W M Q G S L V A Q N Q Q P L A W Y R A

E W M Q G S L V A Q N Q Q P L A W Y R A

tat ttt gat act cct tca ggt gac gaa cca ttg gct ttg gat atg gga tct atg ggt aaa

tat ttt gat act cct tca ggt gac gaa cca ttg gct ttg gat atg gga tct atg ggt aaa

Y F D T P S G D E P L A L D M G S M G K

Y F D T P S G D E P L A L D M G S M G K

gga caa att tgg atc aac gga cag tcc att ggt aga tac tgg act gct tat gct gaa gga

gga caa att tgg atc aac gga cag tcc att ggt aga tac tgg act gct tat gct gaa gga

G Q I W I N G Q S I G R Y W T A Y A E G

G Q I W I N G Q S I G R Y W T A Y A E G

gac tgt aag ggt tgt cat tac aca ggt tca tat aga gct cct aaa tgt caa gct ggt tgt

gac tgt aag ggt tgt cat tac aca ggt tca tat aga gct cct aaa tgt caa gct ggt tgt

D C K G C H Y T G S Y R A P K C Q A G C

D C K G C H Y T G S Y R A P K C Q A G C

gga caa cca act cag aga tgg tac cac gtt cct aga tct tgg ttg cag cca aca aga aat

gga caa cca act cag aga tgg tac cac gtt cct aga tct tgg ttg cag cca aca aga aat

G Q P T Q R W Y H V P R S W L Q P T R N

G Q P T Q R W Y H V P R S W L Q P T R N

ttg ttg gtt gtt ttc gaa gag ttg ggt gga gat tca tct aag att gct ttg gct aaa aga

ttg ttg gtt gtt ttc gaa gag ttg ggt gga gat tca tct aag att gct ttg gct aaa taa

L L V V F E E L G G D S S K I A L A K R

L L V V F E E L G G D S S K I A L A K -

act gtt tct gga gtt tgt gct gac gtt tcc gaa tac cat cct aat att aag aac tgg caa

T V S G V C A D V S E Y H P N I K N W Q

atc gag tct tat ggt gaa cca gag ttc cat act gct aag gtt cac ttg aaa tgt gct cct

I E S Y G E P E F H T A K V H L K C A P

ggt caa act att tcc gct atc aaa ttt gct tca ttc gga aca cca ttg ggt act tgt gga

G Q T I S A I K F A S F G T P L G T C G

aca ttt caa cag ggt gaa tgt cac tct att aat tca aac tct gtt ttg gag aag aaa tgt

T F Q Q G E C H S I N S N S V L E K K C

att ggt ttg caa aga tgt gtt gtt gct atc tcc cct tca aac ttc ggt gga gat cca tgt

I G L Q R C V V A I S P S N F G G D P C

cct gaa gtt atg aag aga gtt gct gtt gag gct gtt tgt tct act gct gct taa

P E V M K R V A V E A V C S T A A -


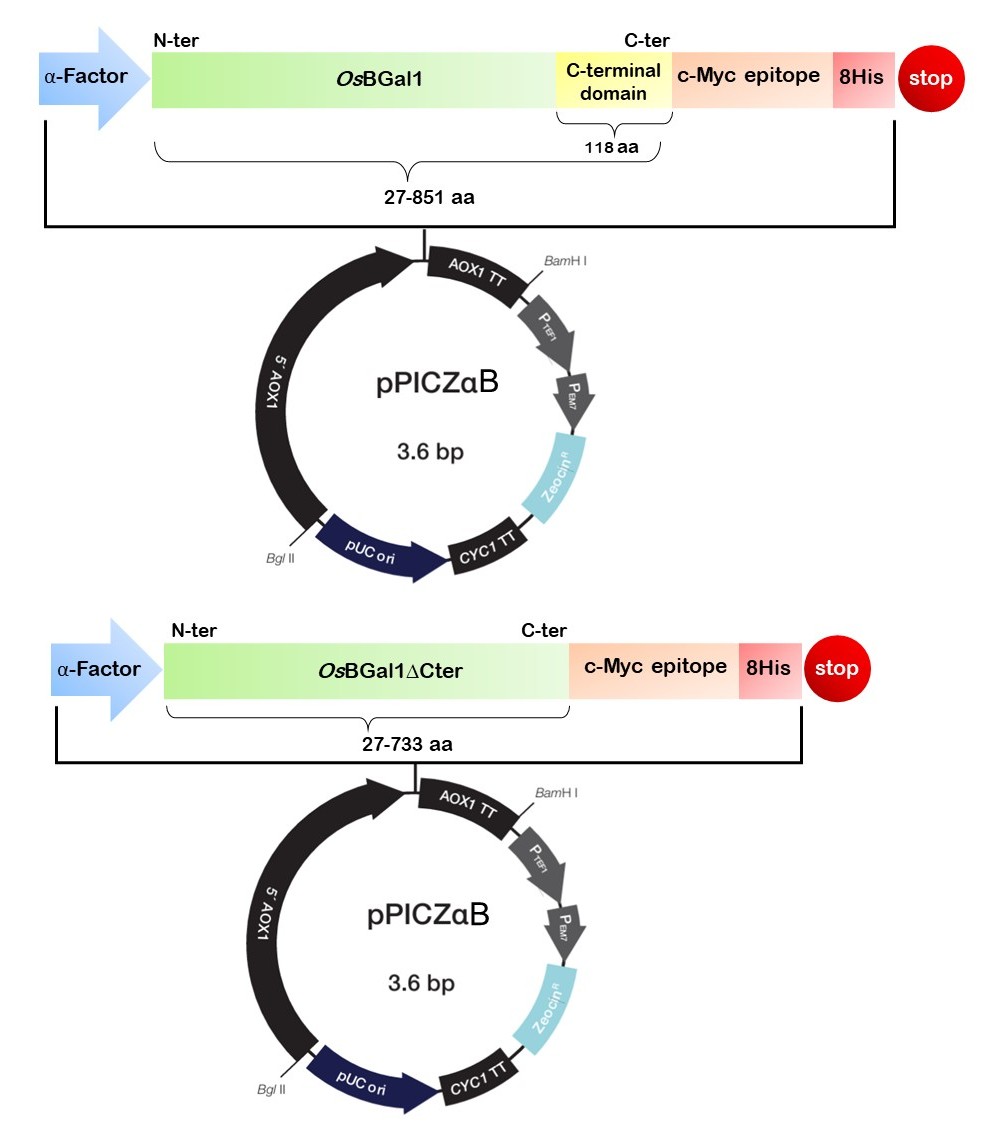


**Fig. S1** Schematic representation of the expression constructs and plasmids used in this study. Full-length *Os*BGal1 comprises amino acid residues 27–851 and includes a C-terminal lectin-like domain (118 aa; residues 734–851), followed by a C-terminal c-Myc epitope and an 8×His tag. The truncated construct, *Os*BGal1ΔCter, lacks the C-terminal domain and consists of residues 27–733, while retaining the same α-factor secretion signal and C-terminal tags. Both constructs were cloned into the pPICZαB vector for expression in *P pastoris*. Relevant vector features are indicated.
